# Exploring the usability of the virtual reality module LEAF CAFÉ: a qualitative think-aloud study

**DOI:** 10.1186/s12877-024-04767-y

**Published:** 2024-02-16

**Authors:** Joyce Siette, Christopher Campbell, Patrick J. Adam, Celia B. Harris

**Affiliations:** The MARCS Institute for Brain, Behaviour and Development, Western Sydney University, Westmead, NSW 2145 Australia

**Keywords:** Virtual reality, Cognition, Older adults, Diagnosis, Dementia

## Abstract

**Background:**

The global healthcare system faces increasing strain from our ageing population, primarily due to the growing prevalence of age-related health conditions such as dementia. While modern healthcare technology offers potential solutions, it frequently lacks user-friendliness for older adults. Virtual Reality (VR) has emerged as a promising tool for diagnosing cognitive impairment, offering innovative solutions where traditional methods may fall short. This study explores older adults’ perspectives on the usability of a newly designed VR module for cognitive assessment.

**Methods:**

During a 100-min session, participants were asked to engage and complete recall and recognition tasks within the VR module (think-aloud approach) and provide feedback upon completion (semi-structured interviews). Audio materials were transcribed for analysis and recordings of the users’ interactions with the module were annotated to provide additional context. These combined textual data were analysed using content coding and thematic analysis to identify themes that reflect how participants used the module’s features and what features are desirable to support that process better.

**Results:**

Participants (*N* = 10; Mean age = 73.3, SD = 7.53, range = 65–83 years) perceived the VR module as user-friendly and endorsed its potential as a cognitive screener due to its engaging and immersive nature. Older adults highlighted three key aspects of the module: the usefulness of the platform’s ability to offer a comprehensive and reliable evaluation of an individual’s cognitive abilities; the need to present concise and relevant content to optimise engagement and use; and the importance of overcoming barriers to support implementation. Suggested game improvements centred on food recognition and adjusting difficulty levels. Barriers to implementation included technology challenges for older adults and concerns about the game’s suitability for everyday scenarios. Participants stressed the need for reliable implementation strategies, proposing locations such as libraries and advocating for home-based screening.

**Conclusion:**

Continued improvements in accessibility suggest that VR tools could help with diagnosing cognitive impairment in older adults. Using a simulated environment to assess cognitive status might fill the gap between current diagnostic methods, aiding treatment planning and early intervention. However, these findings should be approached cautiously, as more research is needed to fully grasp the potential impact of VR tools in this context.

## Background

Globally, the proportion of individuals over the age of 60 will nearly double over the next 30 years, from 12 to 22% [1]. Disease burden of older age is strongly associated with decline in neurocognitive health. As the population becomes increasingly older, the impact of ageing and disease burden is exacerbating the strain on an already overburdened health system [2].

In the past few years, telehealth has emerged as a solution to address the growing need for health services, with increased acceptance of its ability to deliver healthcare and provide health information [3, 4]. It remains important however, that the methods and tools created to either diagnose or treat dementia via telehealth remain highly accessible and approachable, endorsed, and utilised by medical professionals as well as by older people [5, 6].

A plethora of different screening tools have been developed to inspect an individual’s cognitive abilities and track their results over time, in order to formulate a predictive model of a particular person’s cognitive status. A recent systematic review by Abd Razak et al. [7] recommended the Montreal Cognitive Assessment and Addenbrooke’s Cognitive Examination as tools for screening mild cognitive impairment and dementia respectively. However, whilst traditional face-to-face neuropsychological tools, and more recent adaptations of these tools for telehealth [8], have been used to measure cognition, they have several drawbacks. These tests can be time-consuming, expensive and require specialist equipment and trained staff. They may also lack ecological validity, meaning that the tasks do not represent real-world scenarios, and thus are unable to fully capture cognitive function in the context of everyday life [9]. Furthermore, traditional tools may be influenced by cultural and linguistic differences which may impact the validity of results [10].

Technology such as virtual reality (VR) has the potential to address these limitations. VR provides a safe, immersive and interactive environment that can be easily adapted to a range of cognitive assessments and can be completed in a time-efficient manner [11]. Additionally, the use of VR can simulate real-world scenarios to reflect high ecological validity, improving the assessment of cognitive function in context. Finally, VR can reduce the impact of cultural and linguistic factors by exposing users to customised situations that builds a sense of familiarity and relevance that bridges cultural gaps, making it a more universally applicable tool. Indeed, a recent systematic review supported the use of VR as a diagnostic, screening and therapeutic tool for neurocognitive disorders [12], and found that VR tools could predict and support individuals with cognitive impairment, with the ability to consistently track cognition-related outcomes to determine diagnosis. Although VR technology in cognitive assessment has the potential to revolutionize the way cognitive assessments are conducted, little research has explored the acceptability of this form of diagnosis for older adults.

Recent systematic reviews exploring the usability of virtual reality technology among older adults tend to emphasise its application in clinical systems [13, 14], in the context of motor and physical rehabilitation [15, 16], or mental wellbeing [17, 18]. Overall, virtual systems have been rated as usable and feasible, and further usability and user experience pilot studies are encouraged to improve the acceptance and use of VR applications among older adults [14]. Despite the interest and value of VR applications, there is very limited research on the value and acceptability of virtual reality tools for cognitive assessments among older adults. Our study thus aimed to investigate usability and acceptability of virtual reality to screen for dementia in older adults.

## Method

### Study design

A two-phase think-aloud process was used to conduct a task analysis of a VR cognitive assessment module. Participants played the VR module whilst speaking aloud (concurrent think-aloud) and engaged in a subsequent semi-structured qualitative interview to discuss the games’ features, accessibility, and feasibility across their experience (retrospective think-aloud). This study was approved by the Western Sydney University Ethics Committee (H14896) and was conducted between June and August 2022.

### Participants

Our sampling strategy employed a combination of convenience and purposive sampling methods. The target population consisted of older adults aged 65 years and above residing in Australia. Potential participants were identified through local community centres, residential care facilities, and social media advertisement, including online forums (e.g., Facebook). Recruitment efforts included distributing flyers at community events, posting announcements on relevant websites, and establishing collaboration with senior centres. Interested individuals were then screened based on our inclusion criteria, which required a self-reported absence of diagnosed cognitive or vision impairments and willingness to engage with VR technology. Participants were also required to have access to Zoom, the ability to share their screen, and a web browser to play the game. Familiarity with the equipment was not necessary, as the researcher provided guidance throughout the process. This approach aimed to capture a representative sample while considering the logistical constraints and specific characteristics relevant to our study.

Ten participants responded to the recruitment and were sampled, with ages ranging from 65 to 83 years old (*M* = 73.3, SD = 7.53). The majority were female (8/10, 80%). All participants provided informed written consent prior to participating in the study. Each participant received a $20AUD online voucher as reimbursement for their participation.

### Materials

#### Leaf Café assessment module

**Fig. 1:**
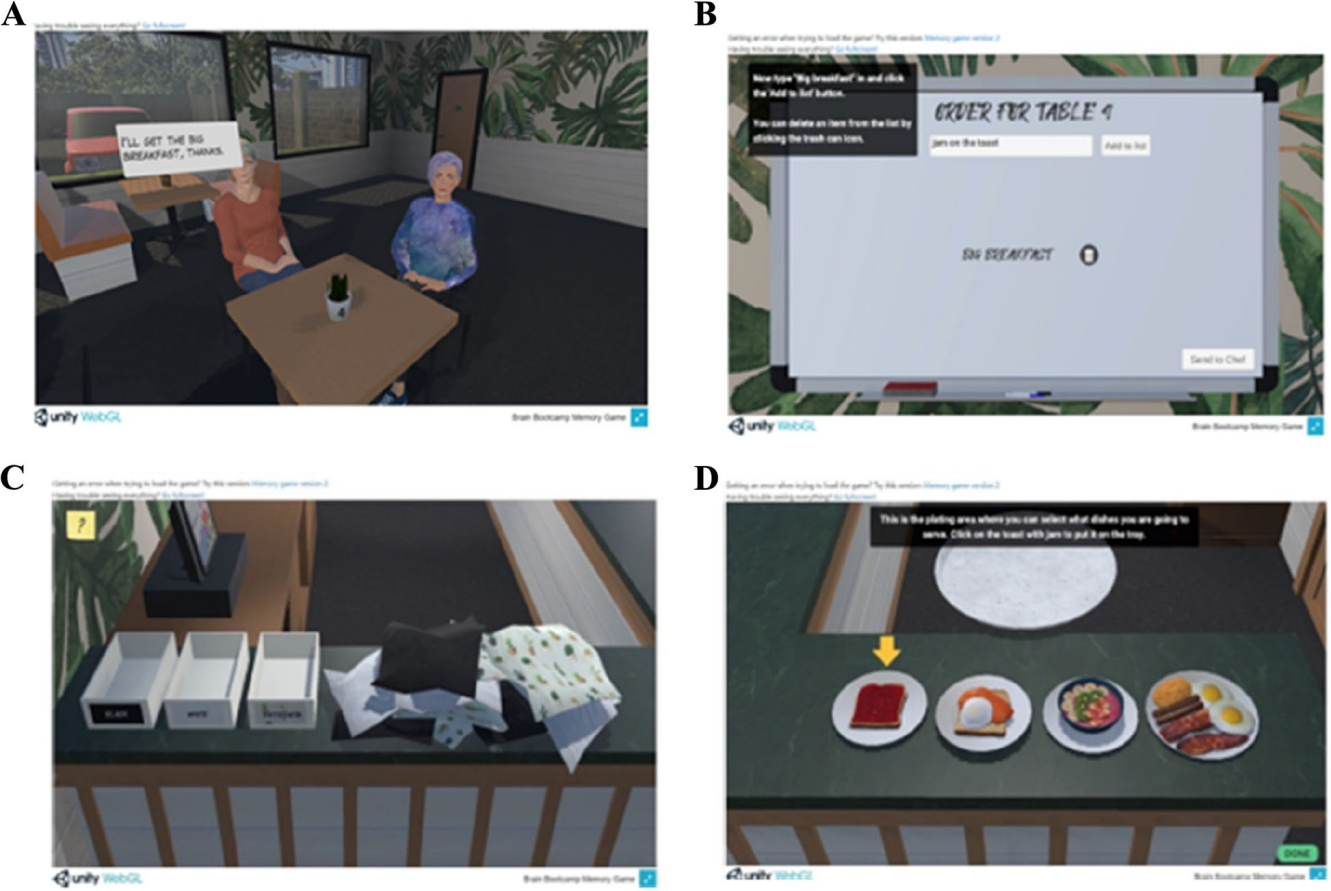
Graphical elements of various scenes in the Leaf Module. **A** Initial presentation of customers at a table. **B** Whiteboard for participant to write down orders. **C** Example of a distraction task. **D** Food counter that participants select food items to serve

Developed using the Unity engine and its WebGL build option, the module could be run on computers with current versions of three major web browsers (Google Chrome, Microsoft Edge, and Mozilla Firefox) [19]. The game places the user in a simulated virtual reality restaurant modelled after Australian cafés where the user takes on the role and responsibilities of a waiter. Each participant was required to attend to restaurant customers as well as complete associated activities assessing participants’ long and short-term memory recall, working memory and executive function. The module commences by introducing four tables with two people sitting at each table (see Fig. 1A). The user is asked to remember the food order from each customer and their table before reporting it to the chef (Fig. 1B). After this, a distractor task is presented (Fig. 1C) to participants that takes roughly 2 min to complete before being asked to provide the orders to the chef again. Lastly, participants are presented with different food options (Fig. 1D) before attempting to serve the food to the right table and customer. There is a total of five levels, with each level increasing in difficulty (Table 1).

**Table 1:** Game difficulty progression

Level	Tables to serve	Customers to serve	Orders
1	1	2	2
2	2	4	4
3	3	6	6
4	4	8	8
5	4	8	16

#### Semi-structured interview

Participants were asked questions before and after the game to extract context and targeted thoughts on their experience. Questions prior to the game focused on knowledge and experiences of screening for dementia and cognitive impairment, and use of VR technologies. Questions following game completion targeted game experience during each stage of the game, feedback and suggested changes. More general questions were asked regarding feasibility and accessibility of a VR screening tool, including questions on technological or physical environment barriers, implementation into a clinical setting, and the presentation of results to users.

#### Think-aloud method

The think-aloud method requires participants to continually speak and air their thoughts aloud whilst they engage in the task. Participants are urged to “keep talking” or “what do you think about what is occurring” if they became quiet for a long period of time to ensure their thoughts are captured [20]. This method is useful as it captures participants’ “in-the-moment” thoughts that are held in the short-term memory, as well as allowing participants to feel heard whilst enhancing and supporting information we obtain from them through the interview questions [21].

### Procedure

Participants were interviewed individually in person or via a Zoom video-call, with each interview averaging 70 min in length (range 27-75 min). Prior to starting, participants either signed a consent form (in-person participants) or were shown a digital copy and asked for verbal consent (Zoom participants). Verbal consent was also requested and given at the start of each recording.

Participants then began to play the LEAF Café module, either to completion or for approximately 45 min. Participants were asked about their thoughts whilst playing the game, such as their ability to understand the game’s instructions, recognisability of the foods, and enjoyment of the graphic design. When participants started to become quiet or respond tangentially, a researcher prompted them on individual game aspects. Once the participant was finished with the game, semi-structured questions were conducted, focusing on their experience of the game and key factors supporting implementation.

#### Data analysis

Talk-aloud and interview data was analysed using thematic analysis [22]. Interviews were firstly transcribed and re-read by CC and JS for familiarisation before independently assigning codes to the transcripts based on preliminary themes. Initial codes were generated, such as ‘tech concern’, ‘visibility’, ‘aesthetics’, ‘item recognition’ and ‘too hard’ and compared between the coders. These themes were discussed and re-grouped into higher-order themes, combining codes deemed similar to each other together to create stronger broad areas for analysis. Disagreements, if any, were then reviewed by CH and discussed in the group. Themes were then collated, to ensure they work in relation to the coded extracts, before being defined and finalised. Data was coded using QSR NVivo 11.

## Results

**Fig. 2:**
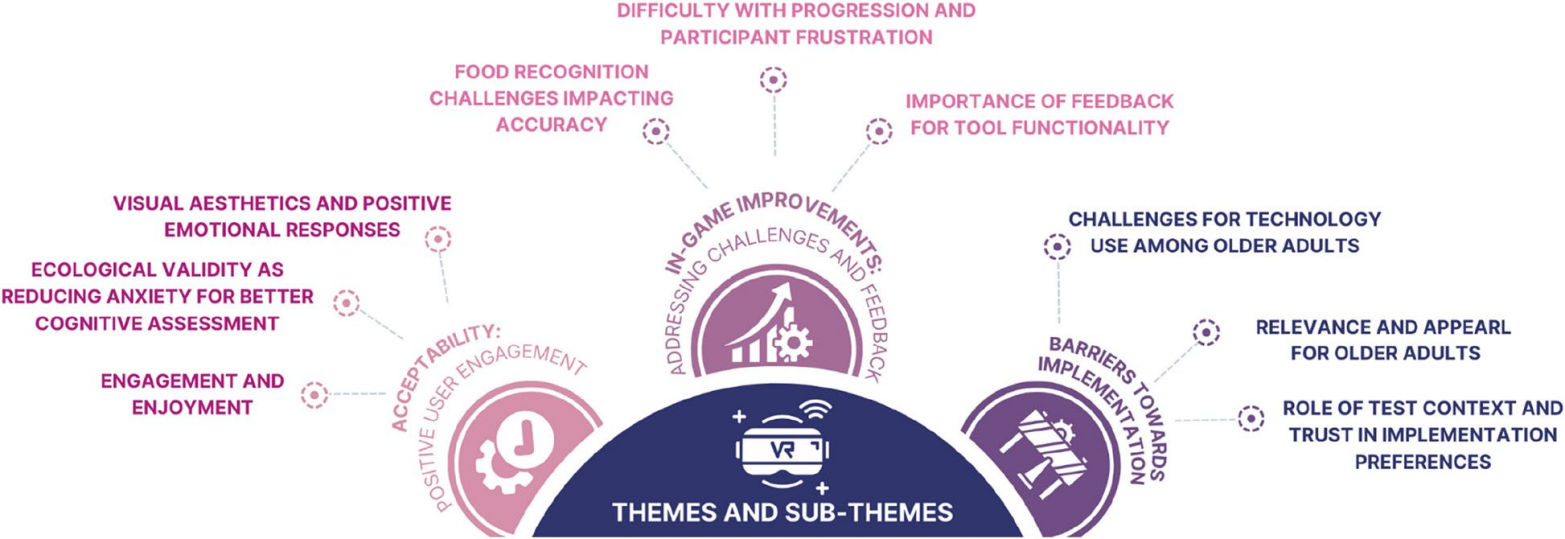
Summary of themes

Thematic analysis generated three broad themes regarding acceptability, in-game improvements and real-world applicability. For each of these broad themes, 3 sub-themes were identified (Fig. 2).

### Acceptability: positive user engagement

#### Engagement and enjoyment

Participants generally described their experience as enjoyable, user-friendly and approachable. They also acknowledged that the experience suited their interests, suggesting its potential effectiveness and appeal as a cognitive screener. Indeed, some participants expressed explicit confidence in the potential of virtual reality platforms.

“It can work for sure.” [Participant 2]

“Yes, it’s definitely something I enjoy and feel it’s tailored.” [Participant 1]

Participants found the module to be a novel and engaging approach to cognitive assessment. Participants also expressed their eagerness to participate, indicating their enthusiasm and willingness to engage with the VR environment, highlighting the appeal and potential enjoyment associated with VR experiences.

“It’s very, it’s very inviting.” [Participant 2]

“I want to play your game.” [Participant 3]

Participants further expressed a sense of relaxation and immersion and appreciated the creation of a realistic and engaging environment that allowed the participant to feel fully present.

“I found it relaxing with the sound on because I felt like I was really there. And just in it.” [Participant 1]

The clarity and quality of the instructions throughout the game was perceived to be well-received.

“They were very clear. The instructions were good. They were great.” [Participant 4]

#### Ecological validity as reducing anxiety for better cognitive assessment

Participants in the study expressed their positive views and highlighted the immersive nature of the technology as engaging and attractive. Participants recognized the potential and usefulness of VR platforms for enhancing existing memory assessments, as they allowed for ecologically valid and standardized evaluations, as well as reducing stress associated with traditional memory tests in the clinic.

“The very act of going to the doctor and talking is somewhat scary for a lot of people. In a game thing, this is somewhere more relaxing for them. They’re immersed in it.” [Participant 1]

“I don’t know what happens if you go to the doctor, and if they do the count back by sevens and all of that stuff. But if it [the result] was looking pretty crook, then I presume you’d be referred somewhere. And I would imagine that wherever you are referred, this game would be useful.” [Participant 5]

This immersive nature further helped to provide a realistic and interactive experience that closely resembled real-life scenarios, whilst permitting participants to experience a sense of comfort and ease in navigating the memory tasks.

“I found it not too stressful, really.” [Participant 3]

“Yeah, it was simple. It was relaxing, you had to do the typing thing, which allowed you time to reinforce what you were trying to remember and doing it at a slower pace. [Participant 1]”

Having cognitive challenges presented through the VR platform was one considered an acceptable method of stimulating an individual’s memory, where the challenge of the task was perceived as stimulating rather than anxiety-provoking.

“I thought it was quite clever. It really stretched one’s memory.” [Participant 5]

#### Visual aesthetics and positive emotional responses

Participants provided positive feedback regarding the visual aesthetics, engagement with various elements, clarity of instructions, and conducive environment provided by the VR experience. There were discussions on the visual quality of the virtual reality experience, particularly regarding positive impression of the graphics and visual presentation of the café environment. Participants expressed their appreciation for various visual aspects including the colours, characters, chef’s demeanour, overall setup, and accompanying pictures. Most participants indicated a positive emotional response, as well as a sense of nostalgia.

“It was graphically pretty good.” [Participant 1]

“I like the colour. I like the people. I like the chef not very angry looking. I like the whole setup. I like your pictures.” [Participant 2]

“I like your game, I like the pictures. I like the food. It looks very nice. It brings me back to my childhood days when you make believe.” [Participant 3]

### In-game improvements: addressing challenges and feedback

This theme relates to participant’s suggestions for improvements within the game and includes three sub-themes, *‘Food recognition challenges’*,*’Difficulty with progression’* and *‘Importance of feedback of results’*.

#### Food recognition challenges impacting accuracy: the importance of familiarity

Some participants reported that their ability to recognise the food was a potential problem that could impact on the accuracy and reliability of the results. Participants often expressed ambiguity to certain food types and required verbal confirmation from the interviewer for the correct dish. They also expressed concern that it could pose as a challenge making the game potentially harder to play.

“There were a number [of dishes] that I wasn’t quite sure what they were.” [Participant 3]

When prompted for further clarification, roughly half of the participants expressed the difficulty was generally with the visual depictions of the food.

“I did know of the foods, I didn’t always get the picture.” [Participant 6]

Furthermore, a few participants also critiqued specific food items that may be less common for an Australian setting and item substitutions to enable better recognition.

“Words like bagel we don’t use much, you know, maybe a bun [instead].” [Participant 5]

“Doing something like Weet-Bix compared to cereal might be more recognisable.” [Participant 5]

These comments highlight the potential value of having tailored content that is recognisable for different cultural groups, to ensure understanding and recognition is not impending accurate task performance.

#### Difficulty with progression and participant frustration

As the game progressed, it was designed to become increasingly challenging, such that participants were required to remember more items of information, and sustain attention for longer periods of time. Participants shared their frustrations and feelings of difficulty during the experience. Half of the participants expressed negative emotions and sentiments such as frustration, loss of control and self-consciousness, which further impacted on their self-esteem.

“I give up, it’s just too hard … Yeah what’s the point?” [Participant 9]

“It was you, you very quickly find yourself losing control. And it’s, I guess, it’s not a good feeling.” [Participant 8]

“And so I’m embarrassed that I’m not up to scratch, you know?” [Participant 2]

Despite these negative feelings associated directly from playing the game, participants understood that this was part of the screening process.

“Yeah, it’s just I guess, the nature of the game, the structure of the game.” [Participant 3]

There were differing perspectives on the appropriate level of difficulty within the VR experience, with some suggesting incremental progression and others advocating for a different approach.

“Maybe you could have two goes at two tables, then at three tables.” [Participant 2]

“I don’t need to think you need to go to this level at all.” [Participant 5]

#### Importance of feedback for tool functionality

This sub-theme describes how participants would desire their results, what they would like to see from them, and subsequent actions based on the results. All participants commented on a strong need for results to be displayed and for feedback to be provided to the participant for the tool to be functional.

“If you don’t get a result, there’s no point playing the game.” [Participant 4]

There was also a consistent desire for instantaneous feedback. Participants recognised the value of self-reflection and feedback in enhancing their learning and performance within the VR environment. Feedback was described to potentially adjust their techniques during the game and improve their results or to limit feelings of frustration.

“Yeah, I think, I think at each stage, how I got it wrong, so that perhaps the next time around, I will pay more attention.” [Participant 2]

“I think maybe at the very least at the end of each stage. Some feedback would be good, if not at the end of the order.” [Participant 3]

Participants further discussed having a system of results that identified their level of cognitive ability, benchmarked results, and when they should be concerned about their results.

“I’d like a score. And then maybe just like a pathology result, you could have ranges that are normal, not normal.” [Participant 4]

“Yes, I would like to be able to track my progress or not, over time.” [Participant 2]

Some participants believe that results on their cognitive ability could facilitate seeking timely medical advice. Participants also spoke about the individualistic nature of the results and how these results could meet individual needs.

“It depends on the person … some people want to know and they want to do something proactive about it, some people might want to look later, when they are a little bit worried.” [Participant 1]

Participants emphasised the importance of having the right to see their medical records and making informed decisions regarding their health. Older adults further highlighted the need for autonomy and informed decision-making in managing one’s own healthcare.

“I think we have a right to see. And then us make that decision whether to go to a doctor or have a copy each one sent to the doctor one sent to you.” [Participant 9]

### Barriers towards implementation: technological challenges and preferences

This theme described perspectives on the feasibility of a VR game being applied in the real world setting and suggestions for effective implementation. It was coded further into three sub-themes *‘Challenges for technology use’*, *‘Relevance and appeal for older adults’* and *‘Role of test context and trust in implementation preferences’.*

#### Challenges for technology use among older adults

This sub-theme identified the critical role of technology, particularly in relation to older adults, and its impact on applicability. Participants discussed the challenges faced by older individuals in using computers and technology, emphasising their limited computer skills, visual and movement impairments, which makes it difficult for them to navigate and access information.

“Old people don’t have computer skills. I’m one of them … But I know my mother and the old people they don’t see well … And all of these moving here moving there, dish dish dish, is just wow, it is tough for them.” [Participant 2]

However, some participants also expressed optimism about the advancements in technology. They believed that technology was continually improving and speculated that future developments could make it more accessible and intuitive for older adults. One participant specifically described the potential for games or applications to become more user-friendly and enjoyable for older individuals.

“I mean, the technology is getting better every day. And you know, we might get to the stage where, you know, it’s it becomes almost intuitive to play these games.” [Participant 3]

Some participants echoed this sentiment, expressing the expectation that technology should be reliable and user-friendly to accommodate the needs and capabilities of older adults.

“Oh, you wouldn’t be able to have that happen [screen did not load]. It’ll have to work better.” [Participant 5]

#### Relevance and appeal for older adults

This subtheme examines participant’s beliefs about the feasibility of the module for assessing cognitive ability for older adults. One participant expressed scepticism about the relevance and appeal of the game concept (i.e., waiting tables) for older adults.

“You know, real life things. It’s not real life to be waiting tables when you’re in your 80s. You know, it’s a game, but would they want to play it? Probably not.” [Participant 4]

Similar thoughts were echoed by other participants, suggesting scenarios that consist of everyday activities of daily living (ADL) such as running errands and creating shopping lists had more value.

“Although I think maybe a more everyday kind of experiencing scenario would be would be helpful … maybe something a list of things to do during the day.” [Participant 3]

The importance of personal agency in healthcare decision-making was also discussed. Participants highlighted their desire to conduct their own cognitive screening and identify any potential concerns or red flags before seeking professional medical advice.

“You know, they want to do their own screening first and see if there’s any red flags or anything like that that they might need to be concerned about before they actually saw a doctor about it.” [Participant 3]

Participants mentioned that there were other physical limitations that might constrain usage of the game, including mobility issues or other medical episodes or diseases (e.g. stroke) that may make partaking in the game difficult.

“If you’ve got, you know, sort of mobility issues with your hands … you’ve had a stroke or something” [Participant 4]

“If a person can’t see the screen properly, because their vision is impaired, it’s not helpful at all.” [Participant 4]

#### The role of test context and trust in implementation preferences

This sub-theme looked more critically at what ways the game should be implemented into a clinical setting and how to best accommodate the target demographic to get the most usage of the screening tool. When prompted on how participants believed this technology may be best implemented, the majority did not feel the doctor’s office would be ideal or feasible and pointed towards other locations, with most participants citing libraries as an ideal location for individual or group testing.

“It takes a long time. So most probably, the doctor’s office isn’t the one, maybe through libraries. Because you’ve got computers in libraries. Maybe the doctor could send you to the library.” [Participant 5]

Responses also included preferences for playing at home compared to a doctor’s office due to comfort and ease. Participants further explained potential benefits of conducting screening at home, such as being at a more convenient time, with their own familiar equipment and setup, that should be considered as the best move for implementation.

“You’d get a better reading as well, I suppose better, you know, because they wouldn’t be so stressed.” [Participant 1]

Feedback also highlighted the importance of leveraging the trust already established with doctors at local clinicals and medical officers. Recognising the significance of this trust, participants suggested it could serve as a key gateway to increasing the use of such a screening tool. 

“If you have one that you are, you know that you have trust in that you, you know, you ask them when you want, when you organise something.” [Participant 7]

## Discussion

This study aimed to explore the ways to improve a user’s experience utilising a VR screening tool and how we can implement virtual reality to effectively screen for cognitive impairment in older people. Our findings collectively support the acceptability and viability of VR platforms as a means of assessing memory in various contexts, offering promising implications for future research and clinical practice. Careful considerations are required to support potential implementation and for future VR screening tools to become even more effective in their purpose.

The acceptability of the VR platform in assessing cognitive ability was influenced by several factors, including perceived usefulness, ease of use, and personal preferences. The positive attitudes expressed by participants towards VR technology as a novel and engaging tool for cognitive assessment align with previous studies [23, 24]. The immersive and interactive nature of VR provided an engaging environment that may enhance motivation and engagement in memory tasks, potentially leading to more accurate and comprehensive assessments.

One possible advantage of an ecological game-based approach to cognitive screening is that it may reduce the anxiety associated with “being tested” on more traditional cognitive measures. For instance, a growing literature on stereotype threat and cognitive performance in older adults indicates that those who are particularly worried about dementia can underperform on neuropsychological tests in clinically significant ways [25]. In this way, balancing task difficulty, sensitivity to changes in performance, and people’s expectations is paramount to ensure that cognitive screening tasks do not induce stereotype threat in older people. Some participants commented on stress associated with the increasing difficulty of the Leaf Café task, and such performance anxiety may induce stereotype threat, if people interpret their failures as indicating the onset of dementia [26]. Future iterations may normalise such stress to reduce any anxiety associated with it and to avoid people negatively interpreting their failures on the task, by emphasising the game-like nature of the task, and instructing participants that the task is designed to get progressively harder and to test the limits of their performance. Moreover, research suggests that stereotype threat has more impact in tasks that are gained-based compared to tasks focused on loss avoidance [27]. The structure of the Leaf Café game may therefore be well suited to avoiding the impact of stereotype threat on older adults’ performance.

Whilst our sample expressed positive attitudes toward VR technology as a novel and engaging tool for cognitive assessment, and recognised its potential to provide immersive and interactive experiences, developing technology based on the needs of the target demographic are more likely to support its usability and accessibility [28]. Indeed, our findings highlights how technological barriers remain one of the biggest hurdles to both the implementation and game-participation process. As expressed by participants, there was a wide range of digital ability and inequality among older adults, which emerged as one of the implementation barriers. Difficulties in navigating the online system and virtual environment may hinder the effective use of these platforms. Ensuring user-friendly interfaces, clear instructions, and comprehensive training programs for both participants and clinicians can mitigate these challenges and enhance its feasibility and usability. Incorporating adjustable visual settings and providing alternative modalities for presenting information can accommodate individuals with visual impairments, ensuring equitable access to VR-based memory assessments.

Contrasting viewpoints highlight the importance of incorporating feedback mechanisms into future VR experiences to optimise users’ learning and engagement outcomes. Our results indicate the complexity and diversity of perspectives surrounding the desire to access and share cognitive results and recognise the importance of personalised approaches in healthcare, understanding that individuals have different needs, fears, and desires when it comes to using their online medical information [29, 30]. Accommodating these individual perspectives can contribute to more patient-centred care and support individuals in making informed choices about their cognitive health management.

To support the further development and refinement of VR screening tools, future research should focus on establishing the reliability and validity of these assessments in comparison to traditional methods. The concerns raised by our sample regarding the accuracy and validity of VR-based assessments highlight the importance of more rigorous validation studies. Whilst there have been some studies comparing the performance of participants on both VR and traditional memory tests [31–33], additional work needs to be done to support the concurrent and predictive validity of VR platforms. Longitudinal studies examining the sensitivity and specificity of VR assessments in detecting memory decline and predicting cognitive outcomes can further establish the clinical utility of these tools.

### Limitations

While efforts were made to recruit a diverse group of participants, our findings may not fully represent the broader population of older adults. Future studies with larger and more diverse samples are needed to validate and extend our findings. By incorporating participants with varying degrees of IT proficiency, we can also better capture the spectrum of experiences and challenges associated with the use of VR tools in memory assessment among older adults.

Furthermore, it is essential to acknowledge the potential for observer bias introduced through the think-aloud scenarios employed during the assessment process. The presence of an observer and the request to verbalize thoughts and actions may have introduced an element of performance anxiety or self-consciousness in some participants [34, 35]. This could have influenced their behaviour and potentially created stress or altered their natural cognitive processes. To minimize this bias, future research could consider implementing more naturalistic and unobtrusive observation methods to capture authentic responses and behaviours during VR memory assessments.

Our study also revealed challenges among older adults in handling computer equipment, particularly arising from problems such as stiffness in hands and other physical or visual impairments. These issues highlight potential limitations in the current VR-based screening method for this demographic. To ensure a more inclusive and comprehensive assessment of cognitive decline and function, alternative or additional methods may need consideration. Technologies incorporating features like eye gaze [36, 37] or voice commands [38–41] could serve as potential alternatives [42], addressing the specific needs and constraints faced by older adults with physical limitations. This approach aligns with the necessity of tailoring assessment tools to the diverse capabilities of the ageing population, fostering a more accessible and effective means of cognitive screening.

Our study also focused on the acceptability and implementation barriers of VR platforms for memory assessment, and thus did not directly measure the clinical effectiveness or diagnostic accuracy of these assessments. Further research is needed to establish the psychometric properties, sensitivity, and specificity of VR-based memory assessments compared to gold standard measures, such as neuropsychological tests or clinical evaluations.

## Conclusion

Our findings support the acceptability and viability of VR platforms as a means of assessing memory in various contexts. The positive attitudes expressed by participants highlight the potential of VR technology to enhance engagement and motivation in memory tasks. However, careful considerations are required for successful implementation, including addressing technological challenges and accommodating individuals with visual impairments. Future research should focus on establishing the reliability and validity of VR assessments, comparing them to traditional methods, and examining their clinical utility in predicting cognitive outcomes. With continued advancements and empirical validation, VR platforms have the potential to revolutionize memory assessment and improve clinical practice in the field of cognitive health.

## Data Availability

Composite data can be made available upon reasonable request to the corresponding author (joyce.siette@westernsydney.edu.au).
